# Factors influencing healthy menopause among immigrant women: a scoping review

**DOI:** 10.1186/s12905-021-01327-z

**Published:** 2021-05-06

**Authors:** Ping Zou, Thumri Waliwitiya, Yan Luo, Winnie Sun, Jing Shao, Hui Zhang, Yanjin Huang

**Affiliations:** 1 School of Nursing, Nipissing University, 750 Dundas Street West, Room 209, Toronto, ON M6J 3S3 Canada; 2 Faculty of Medicine, University of British Columbia, Vancouver, BC V6T 1Z4 Canada; 3 Faculty of Nursing, Health Science Center, Xi’an Jiaotong University, No. 76 Yanta West Road, Xi’an, 710061 Shaanxi China; 4 Faculty of Health Sciences, Ontario Tech University, 2000 Simcoe Street North, UA3033, Oshawa, ON L1H 7K4 Canada; 5 School of Nursing, Zhejiang University, 866 Yuhangtang Road, Hangzhou, 310058 Zhejiang China; 6 Department of Cardiology, Guizhou Provincial People’s Hospital, Guiyang, 550002 Guizhou China; 7 School of Nursing, University of South China, 28 Changshengxi Street, Hengyang, 421001 Hunan China

**Keywords:** Influencing factors, Menopausal transition, Immigrant, Women, Review

## Abstract

**Background:**

Many factors influence the menopausal transition and the complexity of this transition increases with the addition of immigration transition. This review aims to identify the factors that influence the menopausal transition for immigrant women based on ecosocial theory.

**Methods:**

A scoping review of English publications was conducted according to PRISMA guidelines using CINAHL, AgeLine, MEDLINE, PsycINFO, ERIC, Nursing and Allied Health Database, PsycARTICLES, Sociology Database, and Education Research Complete. Thirty-seven papers were included for this review.

**Results:**

The factors which influence the menopausal transition for immigrant women were grouped into three categories: (a) personal factors, (b) familial factors, and (c) community and societal factors. Personal factors include income and employment, physical and psychological health, perceptions of menopause, and acculturation. Familial factors include partner support, relationships with children, and balancing family, work, and personal duties. Community and societal factors encompassed social network, social support, healthcare services, traditional cultural expectations, and discrimination in host countries.

**Conclusions:**

Interventions addressing the menopausal transition for immigrant women should be designed considering different psychosocial factors and actively work to address systemic barriers that negatively impact their transition.

**Supplementary Information:**

The online version contains supplementary material available at 10.1186/s12905-021-01327-z.

## Background

Menopause is reached when a woman experiences a consecutive year of natural amenorrhea without any causal pathological reasons [1]. The menopausal transition is characterized by the presence of menopause-related symptoms and there are often limited options for managing these symptoms. Many factors can affect a woman’s menopausal transition. The complexity of the menopausal transition increases with the addition of an immigration transition as it adds another level of intricacy consisting of migratory, cultural, traditional, economic, community, and social changes. Recently, there has been a great influx of immigrants around the globe. Many of these immigrants are middle-aged women who are concurrently going through their menopausal transition in tandem with their immigrant transition. These two transitions have been explored together in a limited sense. Factors related to a healthy menopausal transition for immigrant women have been explored to an even lesser extent [2]. There is currently a gap in research describing the link between the menopausal transition and the immigrant transition.

Most of the current research on menopause focuses heavily on the biological aspect of this transitional stage of life. Some commonly recognized physical symptoms include hot flashes, sleeping difficulties, and mood swings [1]. However, an immigrant woman’s menopausal transition is also profoundly shaped by the cultural and social transition of immigration since immigrants often face social marginalization. This, coupled with biological changes, can have a profound impact on one’s emotional, mental, physical, and psychosocial wellbeing. Due to the complexity of this transition, it is crucial for researchers, policy makers, and social service providers to understand and target the barriers which negatively impact immigrant women. A psychosocial analysis should be utilized to observe the factors which influence menopausal transition for immigrant women in order to create tailored interventions for support [3].

This review aims to explore the psychosocial factors related to the menopausal transition of immigrant women. The ecosocial theory was used to guide data collection, data analysis, and presentation of the findings. The ecosocial theory is a complex theoretical framework used to examine how social determinants influence disease distribution and encourages critical thinking for an appropriate social intervention of health and wellness [4]. Based on this theory, the psychosocial factors in this review were grouped into the following categories: (a) personal factors, (b) familial factors, and (c) community and societal factors. The examination of the factors influencing the menopausal transition for immigrant women can offer detailed insight into their experiences and inform individuals, families, communities, and the public.

## Methods

### Data source

**Fig. 1:**
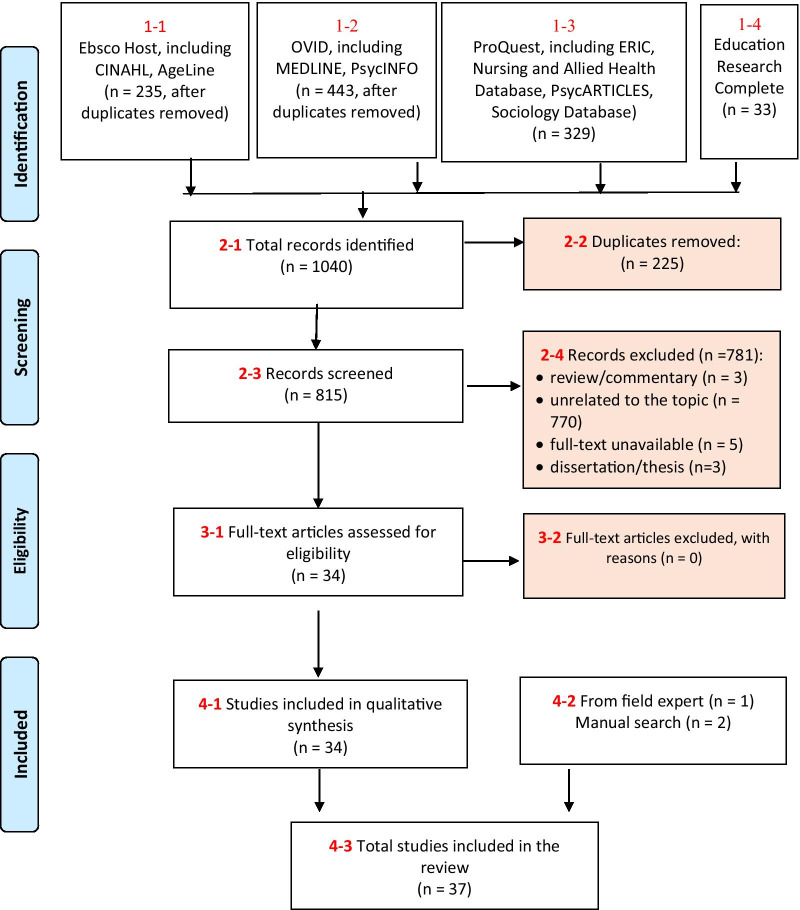
Study selection flow diagram

This review was conducted according to the PRISMA extended guidelines for scoping review [5]. The following medical, health-related, social, psychological, educational, and social science databases were included in the literature search: CINAHL, AgeLine, MEDLINE, PsycINFO, ERIC, Nursing and Allied Health Database, PsycARTICLES, Sociology Database, and Education Research Complete (Fig. 1).

### Eligibility criteria

Studies were included if they: (a) had discussed middle-aged immigrant women (45–65 years) who were in a natural menopausal transition, (b) identified a specific barrier or facilitator to immigrant menopausal health at the individual, familial, or community/societal level, and (c) was written in English. Studies were excluded if: (a) the full text of a paper was not available, (b) immigrant women settled into a new region of the same country, or (c) the article was a review/commentary, or dissertation/thesis.

### Search strategy

The databases were searched using the keywords, (Immigrant* OR Newcomer* OR Settler* OR Migrant* OR Noncitizen* OR Incomer* OR Incoming* OR Foreign*) AND (Menopaus* OR Perimenopaus* OR Midlife* OR Middle-age* OR Middle life). All citations were collected into EndNote reference manager to facilitate the removal of duplicate articles. Based on the pre-established eligibility criteria, the abstracts of all articles were reviewed for relevancy. Then, articles deemed relevant were screened for their full text documents. A manual search was also conducted to gather additional articles.

### Quality assessment

The Critical Appraisal Skills Program Checklists were used to assess all related articles and to classify them as low, moderate, or high quality [6]. Two researchers independently evaluated each article and any discrepancies were discussed until a consensus was reached. Papers rated as low quality were excluded. Thus, all papers included in this review are of moderate and high quality.

### Data extraction and analysis

Based on pre-established criteria, data were extracted by reviewers. A plethora of data, including authors, publication year, sample number and characteristics, research setting, research design, recruitment method, outcomes, and significant findings were extracted. Significant findings were then classified into personal, familial, and community and societal categories. Further facilitators and barriers were identified within each category. Data was organized in Excel and the characteristics of the included studies were presented using descriptive statistics. The findings were also further categorized and summarized using thematic analysis.

## Results

### Characteristics of included studies

Thirty-seven articles were included in this review (Table 1 & Additional File 1): twenty-two (58%) were conducted in the United States of America, five (14%) in Asia, four (11%) in Australia, five (14%) in Europe, and one (3%) in both North America and Asia. Thirty-one (84%) studies independently collected first-hand data and six (16%) studies were secondary analyses of national surveys. Eleven (30%) studies were qualitative, nineteen (51%) were quantitative, and seven (19%) used a mixed-method design. Since some studies shared the same samples, the total sample size of eleven qualitative, nineteen qualitative and seven mixed-method studies were 430, 6786, and 789, respectively.

**Table 1:** Characteristics of included studies

Classification	Number (n)	%
Research design		
Qualitative	11	30% (11/37)
Quantitative	19	51% (19/37)
Mixed-design	7	19% (7/37)
Research methodology		
First hand studies	31	84% (31/37)
Secondary analysis of national survey	6	16% (6/37)
Location		
Asia	5	14% (5/37)
Australia	4	11% (4/37)
Europe	5	14% (5/37)
North America & Asia	1	3% (1/37)
North America	22	58% (22/37)
Research setting		
Medical centre	2	5% (2/37)
Community setting	35	95% (35/37)

### Personal factors

#### Socioeconomic status, income, and employment

Nine studies (24%, 9/37) examined the relationship between socioeconomic status and the menopausal transition of immigrant women. Higher socioeconomic status was linked to better health [7–9]. Higher economic attainment was also correlated with higher education and acculturation, which was further linked to healthy habits. Although immigrants’ education was rarely acknowledged, having a higher pre-immigration socioeconomic status most often led to further opportunities in their new country [10]. Low education and illiteracy were related to psychological complaints, cognitive impairments, increased odds of reporting negative symptoms, and low overall health status [11–15].

Twelve (32%, 12/37) studies discussed career issues related to immigrant women’s menopausal transition. Some women faced challenges when validating their degrees, examinations, and certifications while attempting to find work in their professions. This ultimately led to feelings of being deskilled, low self-satisfaction, low occupational engagement, and emotional distress [16–20]. Most women were confined to low wage occupations and worked under unfavourable labour conditions [8, 21, 22]. Furthermore, occupation related issues, such as unstable employment, unfamiliar working conditions, unpredictable hours, lack of rest time, workplace changes, increased job demands, and the shift from housewife to employee, were also reported as barriers to optimal menopausal health [13, 21, 23]. Women were often unable to focus on their health because they were focused on their careers in order to thrive in a foreign country [23, 24]. Unemployment was also significantly related to the severity of vasomotor and psychological symptoms [15, 17, 25].

#### Physical and psychological health

Nine (24%, 9/37) studies discussed the negative effects of physical ailments and changes on immigrant women’s menopausal transition. Common physical and psychological health complaints, included joint and muscle stress, aching back and neck, [13, 21, 26, 27], cardiovascular symptoms such as palpitations, shortness of breath, and chest pressure [28], sleep disturbances, fatigue, cognitive dysfunction, sexual difficulties [29], obesity, dyslipidemia, depression, and other psychological stressors [13, 17, 22]. In addition, changes in physical appearance, such as graying hair, skin dryness, and sagging arms, also had a negative impact on women’s self-image [24].

Seven (19%, 7/37) studies examined the effect of exercise on immigrant women’s menopausal transition. Few women participated in community sports or regular exercise at country clubs, gyms, or aerobic classes which ultimately negatively affected their health [29]. The most frequently reported barriers to participating in physical activity and exercise were money, language barriers, lack of information regarding physical activity, safety issues, and the perceived lack of space and organized sport programs for this population [20, 30–32]. Furthermore, overweight or obese immigrant menopausal women with weight misperception were likely to report significant barriers to exercise [31]. While the lack of exercise could affect an immigrant women’s menopausal transition, there was a positive relationship between exercise and overall well-being. Immigrants turned to exercise as a method to decrease stress, anxiety, and irritability [16, 30]. There was also a link between physical activity and healthy nutritional habits, which were associated with decreased reporting of chronic conditions [15].

#### Perception of menopause

Eleven (30%, 11/37) studies discussed the impact of perception of menopause on immigrant women’s health. Some women thought that middle-age was the best time in one’s life for stability, maturity, energy, mastery, success, and perfection and they did not think of menopause as a disease [11, 24, 26]. Some cultures normalized and ignored menopause or attributed health symptoms to the natural aging process. Thus, women thought the symptoms they experienced were healthy and normal [8, 10]. Some women managed this transition by taking control of their lives, making their own decisions, managing their treatments, and changing their lifestyle [15, 16]. Consequently, women viewed their menopausal transition in a positive light after they gained control [26]. Having a positive self-esteem served as a buffer against potential unpleasant menopause symptoms [7, 8, 29]. However, some women perceived a lack of power over their own health and viewed the middle-life transition negatively due to its overwhelming responsibilities, association with aging, and loneliness [24, 26, 33]. Women with negative or fearful outlooks towards menopause suffered from prolonged emotional and physical issues through their menopausal transition [16, 24, 34].

#### Acculturation and learning new skills

Six (16%, 6/37) studies examined how acculturation into a new society affected the menopausal transition. High acculturation was linked to norms of expressing health issues and increased awareness of what constituted an alarming medical symptom. This led to an increase in symptom reporting and successful treatment [15, 22, 33]. Acculturation also promoted positive mental health as it decreased social isolation, family, and personal stress [35–37]. Learning new skills, such as English and driving, was beneficial in women’s menopausal transition. Women considered English literacy as an empowering factor as it simplified access to health resources, facilitated socialization in the community, provided opportunities, and decreased stress [8, 38]. On the other hand, reduced English language proficiency made women feel deskilled, inadequate [16, 24], socially marginalized, and stranded within restricted social networks [8, 37]. It also made them feel isolated with limited opportunities to work [26] and become familiarized with the new culture and society [32, 39]. However, being able to drive improved their capacity to access health services, particularly when they started to enter their older years [38].

### Familial factors

#### Relationship with husband/partner

Nine (24%, 9/37) studies examined the divergent effect of differing relationship status of immigrant women and the consequent effect on their menopausal transition. Being married notably increased happiness as it provided an additional source of self-esteem and decreased feelings of loneliness [39]. An early study suggested that immigrant women reported higher marital relationship satisfaction compared with their female counterparts in their migrant country [7]. However, in some cultures, women were typically in a lower social position than men in their families due to patriarchal norms [21]. Such norms made immigrant women’s menopausal transitions more difficult as they were forced to make familial sacrifices [10]. In addition, men viewed their wives differently during and after their menopausal transition, at times seeing them as non-sexual, less womanly, and less attractive. This shift in male attitude was a determinant in domestic violence, extra-marital affairs, and divorce [34]. Divorce and widowhood significantly predicted emotional distress in immigrant women as they felt a loss of support in their lives, making it difficult to manage hormonal changes [11, 16]. Most women recognized that their partner’s support, understanding, and openness to learning about their physical and emotional changes had a positive effect on their health. They reported that their husbands aided them physically, emotionally, and mentally through their immigration and menopause [20, 39]. Contrastingly, as most of the migrant countries encouraged individualistic behavior, immigrant women found themselves feeling decreased dependence on their husbands, which created room for self-growth [9].

#### Relationship with children

Eight (22%, 8/37) studies discussed the beneficial effect of relationships with children on an immigrant women’s menopausal transition. Immigrant women reported that their adult children supported them financially through their immigrant transition and enhanced their adjustment to a new society [39]. Family social support generally moderated the negative relationship between ethnic cultural competence and depression [37]. More specifically, immigrant women who had limited English language abilities benefitted from family members accompanying them to medical appointments [38]. When their children became adults and left home, some immigrant women experienced separation issues and felt loss, depression, anxiety, and sadness [10, 22, 26, 27]. However, some immigrant women cherished the importance of their new identity as grandmothers, identifying this as one of the main positive aspects of aging [29].

#### Competing personal needs and family responsibilities

Fourteen (38%, 14/37) studies examined how familial responsibilities conflicted with immigrant women’s personal needs. After arrival, many women had to assume primary responsibility for their families and children. Thus, women tended to neglect their own needs and gave their menopausal transition less attention than they did to their immigrant and work transition [10, 16, 18, 23, 37, 40, 41]. Some women did not have the monetary and material resources to pursue their own interests or education since they had to work and care for their children [21, 39]. Some women let their husband’s retirement and health take precedence over their own health as they believed they should do their best as mothers and wives [24, 26]. Despite taking the primary role of childcare, women were also expected to equally participate in work and complete all of the household chores [37, 38]. Further, some women’s parents requested their presence in their mother country or they had to care for their aging parents who lived with them, causing stress [16, 19, 42].

### Community and societal factors

#### Social network and support

Five (14%, 5/37) studies examined the impact of social networks on immigrant women’s menopausal transition. Many immigrant women lack social networks and support [21, 30]. This led to difficulties adapting to a new environment [16], a lack of appropriate information on feasible facilities [16, 30], financial issues, emotional distress, and feelings of being stranded [37]. Networking with other women in their new community aided immigrant women as they felt that they were not going through their menopausal transition alone [23].

#### Religious services

Five (14%, 5/37) studies discussed how participation in religious activities positively affected immigrant women’s menopausal transition. Attending religious services on a weekly basis was linked to higher life satisfaction and lower emotional distress [38]. For instance, women believed that their faith helped them consider life in a positive light and helped them get through difficult health situations [18, 39]. Specifically, women believed that prayer, learning about their faith, and participating in religious affairs allowed them to build innate coping abilities in the face of health issues and general life stressors [38]. Furthermore, women believed that their participation in religious events provided them with understanding and guidance which helped them in coping with their menopausal changes [41]. Generally, being part of a religious institution was linked to happiness among immigrant women [39]. It was also noted that immigrants saw religion as the centre of social activity, which ultimately enhanced their sense of belonging in their new community [34, 41].

#### Healthcare services

Ten (27%, 10/37) studies investigated impact of healthcare services on immigrant women’s menopausal transition. Some women who were able to seek aid from their family physician spoke positively about their healthcare experience [38]. However, some women stated that they were confused with all the contradictory information they received from their community healthcare system regarding their menopausal health [16, 19, 26, 43]. Some women felt that physicians ignored their questions [31, 43] and did not take time to get to know them [38]. They also stated that physicians articulated the need for lifestyle changes but did not share information on how to implement such changes [38], prescribed hormone therapy without discussion and information [12], and did not explain procedures, surgeries, and symptoms thoroughly [22, 26, 36]. Women also expressed they were uncomfortable talking about their menopausal health with a male practitioner [31, 43]. In addition, medication and treatment costs created financial strain [38]. Some women did not want to pursue care from mainstream healthcare providers and were able to find alternative health resources in the community, such as herbs and teas [30, 43].

#### Traditional cultural expectations

Eleven (33%, 11/37) studies examined how cultural expectations could spread negative discourse concerning menopausal health. Cultural expectations of impassivity concerning women’s health made women hesitant to talk to their primary healthcare providers about their symptoms, leading to misdiagnoses [18, 24, 26, 29, 34, 43]. Some women minimized the severity of their symptoms as their cultural views posited that their menopausal symptoms were a normal part of the natural aging process [28, 32]. Immigrant women were unable to discuss menopause as freely as Western ones [34] and their symptoms were ignored and endured in silence [10, 16]. In addition, some cultural expectations linked menopause to a decrease in attractiveness, lost value, and uselessness [32], producing a negative attitude towards menopause [27].

#### Discrimination in host countries

Five (14%, 5/37) studies examined the adverse effects of discrimination on the menopausal transition of immigrant women. At times, this population was discriminated against not only in health care settings but also at their workplace [16, 23, 39]. Structural discrimination from healthcare professionals was a barrier to receiving healthcare. Immigrant women did not receive an equal level of care compared to their Western counterparts and healthcare professionals did not recognize their health issues. Immigrant women were not used to the level of assertiveness required with Western providers and, thus, felt uncomfortable when seeking healthcare [40, 43].

## Discussion

### Summary of findings

The findings of this review suggest that an immigrant woman’s menopausal transition is influenced by several personal, family, community and societal factors. Major personal factors include income and employment, physical and psychological health, perceptions of menopause, and acculturation. Familial factors include partner support, relationships with children, and balancing family, work, and personal duties. Community factors encompass social network, social support, healthcare services, traditional cultural expectations, and discrimination.

### Personal factors

#### Socioeconomic status, income, and employment

Our review suggests that employment and income heavily influence the menopause transition for immigrant women. Regular employment and steady income are advantages that improve an immigrant woman’s menopause by decreasing monetary and financial stress. These findings are consistent with the general literature. Disadvantaged socioeconomic status has been significantly linked with poorer health outcomes in all women of all ages [44]. There is also an enduring link between socioeconomic status and health in later life and lower mortality rates. Further, those with higher incomes are more likely to self-report good health and less likely to report depression [45]. Workplace services for immigrant women could potentially mitigate the negative effects of low socioeconomic status on the menopausal transition.

#### Physical and psychological health

Physical and mental ailments have a significant negative impact on immigrant women’s menopausal transition. Physical aspects of health and well-being generally decline during the menopausal transition and many women experience symptoms such as hot flashes and night sweats [46]. Further, the risk for depressive symptoms and disorders is greater during this transition. Depression can result in immense emotional, social, and economic costs from treatment, lost productivity, emotional, and social damage. This risk is greater for African American and Hispanic women [47]. Physical and mental symptoms may be due to hormonal changes but may also be caused by other life circumstances in an immigrant woman’s life. During the menopausal transition, immigrant women may benefit from physical and mood monitoring in addition to an assessment of their situational and environmental surroundings [47]. These actions could lead to earlier interventions to help avoid symptom exacerbation. Our review also elicits that physical activity has the potential to ameliorate physical and mental menopausal symptoms. These findings are consistent with the literature as physical activity has the potential to boost mood and improve some physical symptoms of menopause [48]. It is important for healthcare providers to be aware of these symptoms and their presentation in order to effectively aid women during this transition.

#### Perception of menopause

Our review suggests that menopausal perceptions can greatly impact an immigrant woman’s menopausal transition. This is consistent with the general literature as differing attitudes towards menopause can variably affect a women’s experience. For instance, some women think they will achieve ‘wise women status’ during their menopausal transition and have an increased influence on their family. Further, some women think that qualities such as being feminine and maternal are not given up, rather they are redefined to new roles such as mother-in-law and grandmother. This can have a positive impact on their menopausal transition [49]. Contrarily, some women believe that the primary purpose of women is to be fertile and birth children, thus these women may have a more negative view towards menopause [49]. Perceptions of illness and health are strongly tied to a woman’s culture [50]. These findings imply that empowering women to actively participate in the management of their menopausal symptoms will generally contribute to feelings of control [49].

#### Acculturation and learning new skills

An English language barrier negatively impacts an immigrant women’s transition to a new country and her menopausal transition. If unable to communicate in the host country’s primary language, women may not be able to network, find work, or be readily accepted into their new community. Immigrants who complete training programs or participate in English classes may be able to overcome employment barriers and gain personal benefits [51]. Learning English helps facilitate an occupational identify which increases self-confidence and autonomy. Interventions targeting ongoing language acquisition could improve the menopausal transition for immigrant women [52]. Additionally, having foreign degrees that are not recognized or valued in immigrant hosting countries can negatively impact immigrant women during menopause as this leads to employment barriers and feelings of being deskilled. In general, immigrants’ credentials hold a penalty compared to their host-country-born counterparts. Further, foreign degrees harshly affect visible minority immigrants [53]. This occurs because ambiguities associated with immigrant status release latent biases against immigrant minorities [54]. Policies targeted at recognizing foreign degrees could potentially mitigate these factors [53].

### Familial factors

#### Relationship with husband/partner

Our review suggests that in some cultures, women often feel lower in status compared to their husbands due to patriarchal norms. These findings are consistent with literature and there is limited literature that reports on supportive partners. Stereotyping and gender role formation are developed and instilled in families, where most boys are taught to dominate, control, and present themselves as strong and independent, while girls often try to establish and sustain their relationships [55]. In a traditional nuclear family, the wife is expected to tend to her husband’s needs and take care of the housework which puts a great deal of responsibility on women [56]. These findings imply that there needs to be support in place for all women, including immigrant women, who experience this type of phenomena.

#### Relationship with children

This review supports the finding that children have an important role in supporting immigrant women’s adjustment to a new society which positively impacts women’s menopausal transition. Our findings are partially supported by literature. For instance, a good parent–child relationship is an important motivator for giving and receiving support [57]. Children can bridge the language gap which exists for many immigrant women. This is beneficial for managing health concerns if children are able to accompany women to medical appointments. Our findings imply that children play a more significant role in aiding immigrant women during their menopausal transition than previously discussed in the literature. With this, social services should consider the role of parent–child relationships when designing services.

#### Competing personal needs and family responsibilities

The findings of this review suggest that immigrant women do not have the time to tend to their own needs because of competing responsibilities. These findings are consistent with families in general where women often complete the majority of household chores but are also expected to participate in the workforce and financially contribute to the family [58]. These competing responsibilities result in high stress levels and negatively impact psychological health [58]. Women also have a higher sickness average than men in most Western countries, which is theorized to be related to biological, work, educational, and health factors. This double burden stems from the ‘role strain theory’ which suggests that the mix of multiple roles can increase work strain and adverse health outcomes [59]. This complicated issue is rooted in patriarchal norms. However, organizations could provide extra support to women once they recognize this double burden that many women experience.

### Community and societal factors

#### Social network and support

Our findings reveal that an immigrant woman’s menopausal experience is heavily influenced by their social network and support. A lack of social network and support can lead to an unsafe environment and personal issues. Immigrant women of all ages are found to benefit from co-ethnic communities which facilitate acculturation and community adjustment [60]. For instance, some women received free meals, reduced rent, or gifts of essential household items. However, the nature of this support was not constant, and women were unsure when they could depend on their community [60]. Social support must be available on a continuum to immigrants. Some social support service makers note that immigrants miss out on some services due to language and economic limitations, social isolation, inadequate information from the government, and an affinity to rely on their own social or ethnic group for support [61]. Thus, social support services should consider these factors when designing services.

#### Religious services

Religious services and activities have a positive effect on an immigrant woman’s menopausal transition as they are linked to higher life satisfaction and lower emotional distress. These findings are congruent with the general literature discussing religion and immigrant support. For example, some churches provide language training, assistance with setting up a house, food, furniture, clothing, transportation, health care, housing, and job seeking support [62]. These supports are often inconsistent as most of the service is driven by a small group of volunteers. Additionally, religious services are also agents of cultural and religious assimilation which foster large scale inclusion of immigrants into society [63]. Despite providing both types of support, most religious services tend to meet short-term needs, and the informal nature of these services allow them to dissolve easily. Thus, a proactive approach to supporting immigrants may be more beneficial than the traditional, reactive approach.

#### Healthcare services

This review suggests that immigrant women have unmet healthcare needs during their menopausal transition. In general, female immigrants of varying ages report unmet healthcare needs, while their male counterparts do not [64]. Furthermore, both men and women in the lowest income category have a higher chance of having their healthcare needs unmet than their counterparts in the highest income category [64]. This phenomenon usually occurs due to language differences between the care provider and patient, bias, stereotyping, prejudice, clinical uncertainty of healthcare providers, staff neglect, medication, and diagnostic errors [65]. Further attention needs to be given to immigrant healthcare services and connecting new immigrants to a primary healthcare provider should be done as early as possible in their transition since this is an integral intervention point which may facilitate a smoother transition to their new country’s healthcare system [64].

#### Traditional cultural expectations

Traditional cultural expectations create negative discourse around an immigrant woman’s menopausal transition which can lead to impassivity and shame surrounding menopause. In cultures where menopause is regarded as a natural event, menopause is a sign of aging and is viewed in a positive light [66]. However, some cultures and societies demonize aging as this signals deterioration of the human spirit and lack of energy. These cultural values determine if an immigrant woman feels comfortable and open to bringing up their menopausal transitions and associated symptoms with their healthcare provider [66]. These cultural values, not solely associated with menopause but also with aging, must be taken into consideration when healthcare providers interact with their female immigrant patients [67]. Whenever possible, interactions should be in the patient’s native language with awareness of the specific terms linked to menopause and its associated symptoms. Further, healthcare providers should take precautions to avoid medicalizing and trivializing their patients’ experiences and feelings [67].

#### Discrimination in host countries

This review suggests that discrimination has overwhelming adverse effects on the menopausal transition of immigrant women. These findings are consistent with the general literature. Generally, women experience systematic discrimination in regards to access to power, prestige, and resources. Immigrant women experience even more gender discrimination as they work in informal sectors, occupy lower professional ranks, and receive lower wage jobs while their male counterparts work in professional sectors [68]. Discrimination is also a major source of stress [68]. Furthermore, in addition to sexism, immigrant women face racism. Higher levels of experienced racism is linked to higher suffering of psychological stress symptoms and poorer self-rated health. Overall, discrimination affects the health of women more than their male counterparts [69]. Healthcare providers and immigrant support providers should be specifically aware of these discriminatory practices and the negative impacts it has on immigrant women. Efforts must be made to work backwards and dismantle discrimination’s harmful effects.

### Limitations

This review examined immigration primarily through a Western lens as the search was limited to studies completed in English or translated to English. Most of the literature reviewed investigated the experiences immigrant women in Westernized or developed countries, as opposed to Eastern or developing countries. Thus, our findings cannot be generalized to all immigrant women populations. It is possible that immigrant women who move to non-Westernized countries have different experiences than their Westernized counterparts. There are likely differences in the acculturation process and available resources.

### Significant findings

This review identifies the factors which influence an immigrant woman’s menopausal transition guided by the ecosocial theory. Most notably, we identified several factors at the community and societal level, which serve as major barriers to immigrant women during their menopausal transition. These include systemic discrimination, racism, and sexism. These macro factors impact micro factors for individual women. For instance, sexist ideologies at the macro level negatively impact a woman’s micro interactions and thoughts about herself. Thus, to combat these barriers, individuals and organizations must recognize and be proactive against these factors in order to aid immigrant women through their transition. Moreover, our review identified acculturation and learning new skills as an integral part of the immigrant transition for menopausal women. Further, we were able to identify key relationships and services that aid immigrant women. Thus, our review was able to link together macro and micro facilitators and barriers while also identifying some key novel factors that influence an immigrant women’s menopausal transition.

## Conclusion

The menopausal transition for immigrant women is complex as many women are also concurrently experiencing an immigration transition. This scoping review identified 37 articles that investigated the factors which influence the menopausal transition for immigrant women. From these, three types of influencing factors were identified: (a) personal factors, (b) familial factors, and (c) community and societal factors. Personal factors included socioeconomic status, physical and psychological health, perceptions of menopause, and acculturation. Familial factors included spousal support, relationships with children, and competing responsibilities. Community and societal factors included social network, social support, healthcare services, traditional cultural expectations, and discrimination. It is important that interventions addressing the menopausal transition for immigrant women consider individual, familial, community, and societal factors. Interventions must also actively work to address systemic barriers, which negatively impact this transition. Further research is needed to deepen the understanding of immigrant women’s experiences during menopausal transition, particularly in non-Western countries.

## Supplementary Information

**Additional file 1:** Summary of included studies. All data is contained within the manuscript and the additional file.

## Data Availability

All data is contained within the manuscript and the additional file.

## Abbreviations

PRISMA: Preferred reporting items for systematic reviews and meta-analyses
CINAHL: Cumulative index to nursing & allied health literature
ERIC: Education resources information center
