# Supplementary material for: Factors influencing healthy menopause among immigrant women: a scoping review
Source: BMC Womens Health. 2021 May 6;21:189. doi: 10.1186/s12905-021-01327-z (PMC8101137; doi:10.1186/s12905-021-01327-z)
Supplement: Supplementary file 1 — Additional file 1: Summary of included studies. All data is contained within the manuscript and the additional file. [file 12905_2021_1327_MOESM1_ESM.docx]

**Summary of Included Studies**

|  | **Author (Year)** | **Immigrant hosting country &**  **Women’s background ethnicity/country** | **Research purpose** | **Research method** | **Sample number;**  **Setting;**  **Sampling strategies;**  **Major inclusion/exclusion criteria** | **Significant findings** |
| --- | --- | --- | --- | --- | --- | --- |
| 1 | (Berg, 1999) | Filipino women in the United States of America | To describe the biologic, psychological, social, and cultural dimensions of the perimenopausal transition of Filipino American midlife women | Quantitative;  Cross-sectional descriptive survey;  questionnaire  contained mostly forced-choice questions on demographics, health, menstrual cycles, calcium intake from food  sources, depressed mood, marital relationship satisfaction, and attitudes toward menopause and aging | 165;  Recruited from community churches and social groups;  Included women self-identified as Filipina, American, aged 35-56 years, able to read, speak and write English; not excluded on the basis of health status | - Calcium intake, osteoporosis morbidity, and prevalence of depression were identified as major players in Filipino American’s midlife experience  - The age at menarche nor the number of pregnancies and children born was  significantly related to age at menopause  - Despite high marital relationship satisfaction and positive feelings toward menopause and aging, the  prevalence of depression measured by the screening instrument was high compared with that of  European American samples; this unexpected finding may be attributed to the instrumentation problems sometimes demonstrated by immigrants whose first language is not English |
| 2 | (Im & Meleis, 1999) | Korean women in the United States of America | To extend the previous model of transitions by including the experiences of low-  income Korean immigrant women in the United States during their menopausal transition | Mixed method;  Quantitative analysis was based on data from 119 first-generation Korean immigrant women who engaged in low-status or low-income  work outside their homes; Qualitative study using theoretical sampling method included 21 women. | Total number of participants: 199, Quantitative: 119, Qualitative: 21 (a subset of the 119); Recruited from one large Western city in the United States;  Included women were self-defined first-generation Korean immigrant women 40 to 60 years of age who engaged in low-status or low-income work outside their homes | -This study results in a situation-specific theory of Korean immigrant women’s menopausal transition.  - Women gave their menopausal transition far less attention than they did to their immigrant and work transition  - Menopause was a hidden experience in cultural background  - Women “normalized,” ignored, and endured symptoms. |
| 3 | (Im, Meleis, & Lee, 1999) | Korean women in the United States of America | To describe the experience during menopausal transition of a vulnerable group of women, low income Korean immigrants, | Mixed Method;  Questionnaires, interviews  This study uses the same participant population as (Im & Meleis, 1999) | 119; Recruited using convenience sampling methods from Korean churches and businesses; Included women self-identified first-generation Korean immigrant women aged 40 to 60 years (mean age = 48 years) who engaged in low-status/low-income wage work outside their own homes;  excluded women who could not speak, read, and write Korean | - The types of the symptoms that the women reported were different from those reported by Western women  - The women tended to normalize their symptoms  - Many of these women’s natural experiences with menopause have been medicalized.  - Their symptom experience was influenced by immigration transition and its contextual variables.  - Western researchers often assume that high social status and good self-esteem serve as buffers against potential menopausal symptoms, and women in many non-Western or developing societies do not suffer from menopausal problems because they experience a rise in status at the end of their reproductive life |
| 4 | (Im & Meleis, 2000) | Korean women in the United States of America | To explore the meanings of menopause among a vulnerable group of women—low-income Korean immigrant women, with a focus on how these meanings were constructed with their daily life experiences | Qualitative;  2-hour in depth interviews through audiotaping and field notes; Feminist approach  This is a cross-sectional descriptive study consisting of two parts: quantitative and qualitative. Only the qualitative part about the meanings of menopause is presented in this article.  This study uses the same participant population as (Im & Meleis, 1999), however only utilizes the 21 qualitative participants. | 21;  Recruited using convenience sampling methods from Korean churches and businesses; Included women were self-defined first-generation Korean immigrant women aged 40 to 60 years, who engaged in low-status, low-income wage work outside their own homes (e.g., housecleaning, restaurant work, seamstress), live in the United States for less thatn 10 years; Excluded women could not speak, read, and write Korean | - The shock of menarche, various definitions of menopause, the negative view on middle age as falling down the hill, and other transitions the women were experiencing shaped the nature of their responses to menopause.  - The women were ambivalent about their menopausal transition, welcoming and fearing it  - Nurses should initiate  a dialogue about menopause with diverse populations to support the normalization of women’s menopausal transition and empower them to use culturally appropriate resources. |
| 5 | (Im & Meleis, 2001) | Korean women in the United States of America | To describe how Korean immigrant women tend to describe their work experiences within their daily lives and how they relate their work to the symptoms experienced during midlife | Mixed method; Quantitative analysis was based on data from 119 first-generation Korean immigrant women who engaged in low-status or low-income work outside their homes; Qualitative study using theoretical sampling method included 21 women.  This study uses the same participant population as (Im & Meleis, 1999) | Total number of participants: 199, Quantitative: 119, Qualitative: 21 (a subset of the 119);  119; Recruited using convenience sampling methods from Korean churches and businesses; Included women self-identified first-generation Korean immigrant women aged 40 to 60 years (mean age = 48 years) who engaged in low-status/low-income wage work outside their own homes;  excluded women who could not speak, read, and write Korean | - The symptoms that the women experienced during midlife were influenced by their work experience, which was complicated by their cultural heritage, gender issues embedded in their daily lives, and immigration transition.  - The women were struggling to go through their daily lives while dealing with their symptoms and with the demands of their low status jobs.  - Menopausal experience of low-income immigrant women can be more complicated by other multiple factors because they are more likely to be in vulnerable conditions due to their marginality in the host country, unstable legal status, lack of resources, different cultural values and norms, and their low status jobs. |
| 6 | (Elliot, Berman, & Kim, 2002) | Korean women in Canada | To examine how menopause is experienced by a sample of Korean-Canadian women | Qualitative; semi-structured interviews, Critical ethnographic study | 7; A leader within the Korean community agreed to identify and contact potential  participants. The researchers contacted women who were interested in the study; All participants took part in a face-to-face interview that was structured to encourage dialogue regarding the women’s stories about their experiences of menopause; Included women were 39 to 52 years and lived in a midsized city in Southwestern Ontatio | - The women viewed menopause as a natural process and they used facets of both Korean and Western health practices to manage menopause.  - There was a perception by some of the Korean women that Canadian women are better prepared and manage more effectively because they have more information.  - Korean women suggested that Canadian women might manage this phase of  life more effectively than Korean women because, in Canadian culture, there is  greater openness and willingness to engage in discussions on this topic.  - The participants also felt that the Canadian women had more information be- cause they didn’t face the same language barriers and lack of time or opportunity due to work and family |
| 7 | (Miller & Chandler, 2002) | Former Soviet Union women in the United States of America | To examine relationships among demographic characteristics, acculturation, psychological resilience, and symptoms of depression in midlife women from the former Soviet Union who recently immigrated to the United States. | Quantitative;  Secondary analysis from the Migration and  Health Project, a cross-sectional, descriptive study that examined the impact of immigration and resettlement during midlife on the health and psychological well-being of women from the former Soviet Union. | 200; Women resided in the Chicago metropolitan area; The volunteer sample was recruited from the community through advertisements in a Russian-language news-  paper, announcements in English as a second language classes, flyers posted in neighborhood businesses, and network sampling; Data collection took place in the participants’ homes or in a mutually convenient meeting place; Included women were 45–65 years old, immigrated from the former Soviet Union to the United States after  age 40 and had lived in the U.S. fewer than 6 years; Excluded women had been hospitalized for psychiatric illness within the past 6 years or taking antipsychotic medications. | - Very high scores on the depression scale compared to U.S. norms.  - Older women, and those reporting greater demands of immigration, had higher scores on the depression scale.  - Lower depression scores were found for women reporting greater English usage and resilience  - The study findings indicate that midlife women from the Former Soviet Union report many symptoms of depressed mood, and they experience these symptoms frequently during their first few years in the U.S. |
| 8 | (Im, 2003) | Korean women in the United States of America | To examine cultural influences on symptoms experienced during menopausal transition of Korean women in South Korea and Korean immigrant women in the United States. | Mixed methods;  First study: questionnaire on sociodemographic characteristics and symptoms were given to the women in person.  Second study: Quantitative analysis was based on data from 119 first-generation Korean immigrant women who engaged in low-status or low-income  work outside their homes; Qualitative study using theoretical sampling method included 21  women. | First study:  426;  Convenience sampling methods;  Recruited from Churches and cultural centres;  Inclusion women were Korean who were born and living in South Korea, 40-60 years, and did not report any diagnosed health problems  Second study:  119; Recruited from Churches and businesses;  Inclusion criteria: self-identification as first-gen Korean immigrant female, 40-60 years without any diagnosed  health problems. This study uses the same participant population as (Im & Meleis, 1999) | - Korean women in South Korea tended to report more symptoms than Korean immigrant women in the United States  - Types and severity of prevalent symptoms were also found to be different between the two groups.  - Historical and sociocultural contexts of women’s daily experiences (e.g., cultural transition, influences of pharmaceutical industries,  marginalized status as immigrant women) are important in understanding women’s menopausal experiences.  - There is a need to consider diversities and complexities of women’s menopausal experiences even among women with a common cultural heritage when assessing and providing health care for menopausal women, and in planning and conducting research on menopausal women. |
| 9 | (Miller & Gross, 2004) | Former Soviet Union women in the United States of America and Israel | To compare depressed mood between midlife women from the FSU who reside in the United States and Israel, controlling for demographic and health characteristics | Quantitative  Descriptive secondary analysis of data collected independently in the United States and Israel in 1997–1998.  Data for the U.S. sample are from a cross-sectional, descriptive study that examined the impact of immigration on health and psychological well being of 200 women from the FSU who resided in a large Midwestern metropolitan area in 1997–1998.  The data for the Israeli sample were collected  as part of a national telephone survey that included women age 22 and over who were permanent residents of Israel. | 72 community dwelling women; 36 from the USA, 36 from Israel  Included women in the United States were 45–65 years, and residence in the United States for up to 6 years; Women were recruited through community advertisements, English classes, and network sampling  The data for the Israeli sample were collected  as part of a national telephone survey that included women age 22 and over who were permanent residents of Israel; Age matching  was elected to equalize the number of women from the two samples, and because the samples differed most substantially in age and length of time since immigration.  Israeli women who were born in the former Soviet Union and interviewed in the Russian language were matched by age (within 5 years) and number of years since immigration (within 3 years) to women in the U.S. study group. This method yielded 36 matched pairs. | - Living in the United States, having lower self-reported health status, and having arthritis predicted higher depression scores.  - Future cross-national interdisciplinary research should be directed toward identifying specific contextual factors that will guide interventions and influence health policy for new immigrants.  - Do not know which host country factors, such as attitude toward immigrants, financial support mechanisms, community-based language and acculturation services, health care accessibility and utilization, density of co-ethnics, and informal support networks, interact with personal characteristics and contribute the most to immigrant adjustment. |
| 10 | (Miller, Chandler, Wilbur, & Sorokin, 2004) | Former Soviet Union women in the United States of America | To examine relationships among acculturation, depression, and cardiovascular risk factors in midlife women from the former Soviet Union and identifies factors predicting Framingham Risk Scores | Quantitative; Longitudinal study, Questionnaires | 218; Recruited from community centres though radio, community; This volunteer sample of women resided in urban and suburban neighborhoods of a large, Midwest metropolitan area; Data collection took place at participants homes or in groups; Included women were 40-70 years, immigrated to the USA from FSU less than 8 years before recruitment, married, had at least one child in the USA | - The leading risk factors were obesity, dyslipidemia, and depression.  - Older women had lower American Behavioral Acculturation subscale scores, higher Russian Behavioral Acculturation subscale scores, and higher depression scores.  - Length of residence was significantly correlated with American behavioral acculturation but not Russian behavioral acculturation  - Baseline body mass index, both acculturation scores, and depression scores predicted Framingham Risk Scores after 1 year, but serum glucose did not. |
| 11 | (Miller, Sorokin, Wilbur, & Chandler, 2004) | Former Soviet Union women in the United States of America | To examine symptoms of depressed mood in relation to age, menopausal status, and length of residence in the United States in midlife women who are recent immigrants from the former Soviet Union. | Quantitative; cross-sectional analysis, Questionnaires  Data for this cross-sectional analysis are from the Chicago Health after Immigration (CHAI) Project, a longitudinal study of the influence of acculturation, family adaptation, and health behavior on post-immigration health status and psychological well-being | 220; This volunteer sample was recruited from the community through advertisements in a Russian-language newspaper, English as second language classes, posters in neighborhood businesses, and network sampling. The women resided in both urban and suburban neighborhoods; Data were collected individually in the participants’ homes or in small groups that met in community meeting places; Included women who were 40 –70 years old, had emigrated from the former Soviet Union fewer than 8 years prior to enrollment and who were married with at least one child living in the United States. | - Regression analysis indicated that even when use of antidepressant medication was held constant, age and residence in the United States were significant independent contributors to CES-D score: women who were older, had lived fewer years in the United States, and those who took antidepressants had higher CES-D scores  - Cultural and immigration-related explanations for high scores on the depression scale are suggested. |
| 12 | (Esposito, 2005) | Hispanic women in the United States of America | To examine immigrant Hispanic women’s and providers’ assumptions about and expectations of healthcare encounters in the context of menopause | Qualitative;  Interviews;  Descriptive focus group study | 56: 40 women participated in 4 Spanish-language focus  Groups, 6 healthcare providers, 5 nurse practitioners and  1 physician, took part in a fifth English-language focus group; Focus group interviews were held on the grounds of a medical center located in a predominantly Hispanic community; Convenience and snowball sampling; Included women aged 39-62 years and who were fluent in Spanish | - Providers believed that menopause was an unimportant health issue for immigrant women and was overshadowed by concerns about high-risk medical problems, such as diabetes, heart disease and HIV prevention.  - The women expected a healthcare encounter to be patient centered, social, and complete in itself.  - Providers expected an encounter to be business-like and one part of multiple visit care.  - Language and lack of time were barriers cited by all.  - Dissonance between patient-provider assumptions and expectations around issues of healthcare leads to missed opportunities for care. |
| 13 | (Miller et al., 2006) | Soviet Union women in the United States of America | To determine the effects of acculturation, social alienation, personal and family stress, and demographic characteristics on depressed mood in midlife immigrant women from the former Soviet Union | Quantitative;  Demographic characteristics, including age, length of time in the United States, education, ethnicity/religion in the former Soviet Union, and the republic from which the women had emigrated, were obtained by self-report data.  The study reported here involved a cross-sectional  analysis of baseline data from a study of the impact  of acculturation on health and behavior change in midlife women from the FSU. | 226; The sample was recruited from the community through Russian radio and newspaper advertisements, flyers in neighborhood businesses and clinics, announcements in English as second language (ESL) classes, and network sampling; Sample of women resided in urban and  suburban neighborhoods of a large Midwest  metropolitan area;  Inclusion women were 40-75 years, immigrated to the USA from the FSU within 8 years prior to recruitment, married, had at least one child living in the USA | - Higher acculturation levels promoted mental health indirectly by reducing social alienation and, subsequently, lowering family and personal stress, both of which had direct relationships to symptoms of depression  - School social support mediated the positive  relationship between host culture acculturation  and mental health  - Family social support, on the other hand, mediated the negative relationship between ethnic cultural competence and depression  - Perceived family and personal stress were both  strongly affected by social alienation and were, in  turn, found to be important direct predictors of  depressed mood |
| 14 | (Hafiz, Liu, & Eden, 2007) | Indian women in Sydney, Australia | To examine the experience of menopause in Indian women in Sydney, and the relationship between sociodemographic factors and menopausal symptoms, and also to explore the cultural context | Mixed method;  Interview with 29-item Menopause-Specific Quality of Life questionnaire. | 203; Participants were interviewed in collaboration with the Multicultural Health Unit of the South-Eastern Sydney Area Health Service as well as through four ethnic  associations in the area; Each participant was interviewed once by experienced bilingual health workers from the Multicultural Health Unit, either by phone or face-to-face, as they preferred; Included women aged 45–65 years; Excluded women with pregnancy and breast-feeding, hormone therapy, and a history of drug and alcohol abuse. | - Lower scores of menopause symptoms indicate that Indian women have fewer complaints of symptoms and a positive attitude towards menopause.  - Somatic symptoms are multifactorial in nature and could be because of health problems associated with ageing, midlife crises and cultural influences.  - Further detailed studies could examine the important relationship between cultural lifestyle factors and climacteric symptoms. |
| 15 | (Liu & Eden, 2007) | Chinese women in Sydney, Australia | To investigate the menopausal experience of Chinese women living in Sydney and to explore the prevalence of symptoms, and the relationship between the frequency of symptoms and various socio-demographic factors. | Quantitative; A cross-sectional survey was conducted. The menopause-specific quality of life (MENQOL) questionnaire was used to collect information on menopausal symptoms. | 310; Eight locations, including Chinese churches, Migrant resources centres, and Adult Migrant English Services, were visited. Completed by either face-to-face or telephone interview; Included women 45-65 years; Excluded women who were pregnant, breast-feeding, users of hormone therapy (hormonal contraception, hormonal replacement therapy), or who had a history of drug or alcohol abuse | - Chinese women living in Sydney report fewer vasomotor symptoms compared with Caucasian women.  - Menopause  was still experienced negatively, especially in its impact on sexual function and muscular–skeletal symptoms. |
| 16 | (Yang et al., 2007) | Korean women in the United States of America | To investigate the level of leisure-time physical activity (LTPA) among midlife Korean American women and to determine the relationships of LTPA with individual characteristics and behavior-specific cognition and affects. | Mixed Methods;  Questionnaire, cross-sectional descriptive study | 152; recruited potential participants by posting flyers on public bulletin boards in the community (for example, in local Korean grocery stores or Korean ethnic churches in the Central Texas area). Additionally, informal leaders in church activity groups were consulted for help in attracting candidates to the study; Included women self identified original ethnicity as Korean women or Korean American women, ability to read and write either Korean or English, 45-65 years, absence of physical illnesses or disabilities that would limit daily physical activities | - The level of acculturation was not significantly related to the amount of physical activity.  - After age, level of acculturation, education, income, and marital status were controlled, LTPA was significantly associated with perceived benefits and barriers and social support.  - Physical inactivity, which was common in this group of immigrant women, warrants further rigorous investigation to determine the dynamics of the women’s involvement in more physical activity. |
| 17 | (Liu & Eden, 2008) | Greek in Sydney, Australia | To investigate the menopausal experience of Greek women as part of a wider survey of four immigrant groups living in Sydney, Australia (the others being Indian, Arabic, and Chinese) and to examine the relationship between common symptoms and various sociodemographic factors. | Quantitative;  Interviews  29-item Menopause-Specific Quality of Life questionnaire | 217;  30% of the respondents were interviewed by  phone through a Greek general practitioner clinic, and the rest were face-to-face personal contacts or approached through church groups and social functions.;  Through personal contact, church groups, social function;  Included women were 45-65 years and were Greek living in Sydney | - Significant findings were found for retired Greek women in the pre- or perimenopausal stage and with psychosocial symptoms; there was also an association between postmenopausal obese and married women with sexual problems  - High rates of physical  symptoms that were reported, particularly feelings of fatigue and lack of stamina, and the considerable rates of psychosocial complaints were also found |
| 18 | (Remennick, 2008) | Native and immigrant Jewish women in Israel | To compare the perceptions and practices of health between native Israeli women and recent immigrants from the former Soviet Union. | Mixed methods; Structured questionnaire, followed by personal interviews | 315: 158 Native Israelis, 157 Russian Immigrants;  Recruited through their workplaces - employee records of three white-collar work places located in Central  Israel, which represented three different economic sectors (medicine,  insurance, and telecommunications); Included women were 45-65 years, of Ashkenazi, that is, European, origin and middle-class background | - Health interventions aimed at middle-aged women should be specifically tailored, accounting for different cultural constructions of aging and menopause. |
| 19 | (Resick, 2008) | Russian women in the United States of America | To explore the meaning of health among midlife Russian-speaking women from the former Soviet Union. | Qualitative; interview, hermeneutic phenomenological design; demographic questionnaire followed by a semi-structured list of questions | 12; Purposive and snowball sampling methods; The majority of the interviews took place in the homes of the participants; three women were interviewed in the workplace; and one interview took place in a private dining area; Included women were 40–61, who also spoke English and had migrated to the United States after 1991. | - Although health was less of a priority during the immigration process, the women valued and were knowledgeable about health, participated in self-care practices, trusted their own abilities to make self-care decisions, and sought health-related information.  - This is a vulnerable population at risk for the onset of chronic medical conditions associated with the process of aging, past exposures, the tendency to avoid health screening, and current stressors related to immigration and family responsibilities.  - Implications include the need for interventions to build trust, assess self-care practices, and understand values and beliefs concerning health screening. |
| 20 | (Binfa, Robertson, & Ransjo-Arvidson, 2010) | Chilean women in Sweden | To examine how Chilean immigrant women living in Sweden perceived and related their life situations and health status during midlife to their migration experiences | Qualitative,  Focus group discussions and three in-depth interviews were performed through content analysis in order to capture experiences that could be more difficult to explore with focus group discussions | 21;  Community location;  Snowball sampling technique (a Chilean women working with immigrants in Sweden recruited the first participants), the rest of recruitment was done through a purposive sample procedure; Included women aged 40-60 years, live in Stockholm for at least 15-20 years | - three major themes emerged: (i) Chilean women’s reflections about migration and resettlement; (ii) Health during midlife; perceptions of Chilean women living in Sweden and (iii) Strategies to manage their lives and to gain social acceptance and position  - Women found it difficult to cope with their new situation – living with earlier trauma while also assimilating into Swedish society  - Some had heartbreaking loss of families and social networks in Chile  - There was concern about ignorance among the Swedish authorities regarding the political situation in Chile – no treatment for trauma or psychological support was available  - Most women felt that they were deskilled (had problems with validating degrees, examinations, certifications)  - Carrying the main responsibility made them ignore their own health until their inevitable symptoms affected their daily lives, limiting their working capacity |
| 21 | (Lerner-Geva, Boyko, Blumstein, & Benyamini, 2010) | Jewish, Jewish immigrants from Russia, Arab women in Israel | To examine differences in symptom clusters among women in midlife from different cultural origins and to identify sociodemographic, lifestyle, and health characteristics that could account for the differences between the cultural groups in symptom reporting | Quantitative;  Structured questionnaire was created to collect information on women’s physical and mental health at ages 45–64 in Israel and to examine a wide range of health behaviors, health beliefs, and attitudes to preventive behavior  Secondary analysis from the Women’s Health in Midlife National Study (WHiMNS) which was designed to sample three main subpopulations of women in the relevant age group: Jewish residents born in Israel or who immigrated to Israel up to 1989 (LTR), Jewish immigrants from the former Soviet Union who arrived after 1989 (immigrants) and Arab Israelis. | Israeli women aged 45–64 were randomly selected according to age and population strata of three groups: long-term Jewish residents (LTR), Jewish immigrants from the former Soviet Union, and Arab women (mostly Israeli-born). Interviews were conducted with 540 LTR, 151 immigrants, and 123 Arab women; data was collected at the participant’s homes; The WHiMNS sample consisted of women living in Israel as of January 2004, randomly selected from the  National Population Registry, stratified by 5-year age groups and population group (LTR, Immigrants, and Arabs). Two hundred women were randomly selected in each of the 12 age and group strata, with the aim of reaching 600 LTR, 200 immigrants, and 200 Arab women. To arrive at a maximal number of interviewees, replacement of those who refused or were not  located was allowed | - Cultural group is an independent predictor of each of the three menopausal symptom scales.  - A possible explanation for the lower reporting of symptoms among Arab and immigrant groups is that they differ from the LTR in level of acculturation and attitudes toward menopause. |
| 22 | (Boral, Borde, Kentenich, Wernecke, & David, 2013) | Turkish women in Berlin, Germany | To examine and compare common perceptions of menopausal symptoms among migrant women from Turkey in Berlin | Quantitative;  Survey with a structured questionnaire in the German and Turkish languages, which contained questions about their experiences with the menopausal phase and related symptoms (Menopause Rating Scale II), menopausal hormone  therapy, and sociodemographic, psychosocial, and migration-related aspects. | 963;  Random and snowballing sampling;  Included women aged 40-60 years, Excluded women who had undergone hysterectomy, bilateral ovariectomy, or antiestrogen therapy | - Experience of migration and the menopausal phase are both not obligatorily problematic in women, however, it is worth discussing whether the  concomitance of difficult socioeconomic aspects and a difficult migration-related situation might impact the menopausal transition and possibly intensify the perception of complaints  in this phase, as it is likely to impact the overall well-being of an individual  - Difficult socioeconomic conditions and a demanding migration-related situation might add up to a higher psychosocial burden in midlife migrant women |
| 23 | (Hyejeong & Pak, 2013) | Korean women in the United States of America | To examine the experience of five Korean immigrant women in midlife, specifically the cultural influences on their psychological and spiritual changes in the process of integrating two different cultures | Qualitative;  Semi-structured interviews with Life story method; Data was analyzed by employing within-case analysis and cross-case analysis | 5; Participants attended churches; Included women were born and raised in Korea at least until they finished formal education, immigrated to the USA as an adult and have lived in the USA longer than 20 years, Christian women in their midlife who were recognized as devoted to their spiritual maturity, currently residing in Southern California | - Each participant’s spiritual path and God’s image evolved as a result of multiple factors, which include early parent-child relationships, gender socialization, marital relationships, adaptation to the cultural norms of the immigrant society, and their stage of life.  - Initially, the participants focused on communal spirituality as they adopted the individualistic host culture, and then progressed towards private spirituality as they adopted the individualistic host culture. |
| 24 | (Irvin et al., 2013) | Korean women in the United States of America | To examine the relationship between prevalence of osteoporosis and milk consumption, and their relationship with acculturation among a representative sample of immigrant California women of Korean descent. | Quantitative; telephone surveys | 590; Included women were of Californian women of Korean descent, they had to be either pre or post menopause | - Acculturation was related to higher prevalence of osteoporosis among postmenopausal, but not pre-menopausal Korean women in California.  - Future research should include larger cohorts, objective measures of osteoporosis, other sources of calcium specific to Korean cuisine, and assessment of bone loading physical activity  - Though acculturation was related to higher milk  consumption during the younger life periods, it was not related to current consumption. Immigrants from Korea are exposed to more milk through the increased availability in stores and broader social acceptance in the US. |
| 25 | (Perez-Alcala, Sievert, Obermeyer, & Reher, 2013a) | Latin-America women in Madrid, Spain | To examine age at menopause in relation to demographic and life style factors among  Latin-American immigrants to Madrid and their Spanish counterparts | Quantitative; closed and open ended questions, Questionnaire by face to face interviews  Secondary analysis from 2 surveys, the Decisions at Menopause Study in 2002-2003 and Latin-American immigrants to Madrid (2010–2011)  The Spanish survey was carried out in a randomly selected sample of 300 women aged 45 to 55 years and residing in Madrid, Spain in 2010-2011. The survey was conducted in Madrid in 2002-2003 as part of The Decisions At Menopause Study | 484; Included women aged 45 to 55 years and residing in Madrid,  Spain; Excluded women  with with a history of hysterectomy, oophorec-  tomy, or chemotherapy and women currently using hormone therapy | - Early life events, including place of birth, and later life  events, such as timing of migration, were associated with age at menopause.  - This study highlights the importance of taking into account differences in the age of onset of menopause in the multicultural population of Madrid when considering the health of women at midlife and beyond. |
| 26 | (Perez-Alcala, Sievert, Obermeyer, & Reher, 2013b) | Latin-America women in Madrid, Spain | To examine the determinants of hot flashes and night sweats within immigrant and local populations in Madrid, Spain. | Quantitative; closed and open ended questions, Questionnaire by face to face interviews  Secondary analysis from 2 surveys, the Decisions at Menopause Study in 2002-2003 and Latin-American immigrants to Madrid (2010–2011)  The Spanish survey was carried out in a randomly selected sample of 300 women aged 45 to 55 years and residing in Madrid, Spain in 2010-2011. The survey was conducted in Madrid in 2002-2003 as part of The Decisions At Menopause Study  This study uses the same participant population as (Perez-Alcala, Sievert, Obermeyer, & Reher, 2013a) | 484; Included women aged 45 to 55 years and residing in Madrid,  Spain; Excluded women  with a history of hysterectomy, oophorectomy, or chemotherapy and women currently using hormone therapy | - Compared with Spanish women, Latin-American women were less likely to report hot flashes after demographic variables and menopause status were controlled for.  - The same was not found for night sweats and for both symptoms combined.  - Determinants of hot flashes differed from determinants of night sweats. |
| 27 | (Choi, Kushner, Mill, & Lai, 2014) | Korean women in Canada | To examine midlife and older Korean immigrant women’s experiences following their immigration to Canada | Qualitative;  Interview (focused ethnography) | 15;  Recruitment through flyers, contact with church pastors, and cultural broker;  Included women who emigrated from Korea, permanent resident or Canadian citizen, had been living in Canada for at least 5 years, were 50 years or older at the time of the interview, were living at the study site, were able to understand and speak Korean or English | - In coming to Canada, women focused on caring for their children and often  sacrificed their personal dreams  - They had to be employed to support their families, and received support from family and government  - Women participated regularly in a Korean Church and drew on their Christian faith to ease their adjustment. They retained hopes for the future including good health and a better life for their children.  - Most women indicated that it was difficult to integrate into Canadian society but they never gave up on their adjustment to a new culture |
| 28 | (Hinrichsen, Wernecke, Schalinski, Borde, & David, 2014) | Chinese women in Germany | To detail the differences between the occurrence of menopausal symptoms in German women, migrant Chinese women in Germany and Chinese women in their native country. | Quantitative; set of questionnaires  surveying socio-demographic data, use of hormone therapy, migration/ acculturation, MRS II and other areas; cross-sectional studies where the 3 groups of women were surveyed on the topic of menopause and hormone therapy using an evaluated set of questionnaires | 1000 German women and 852 Asian women;  Included women were aged 45– 60 years at the time of the survey, nationality (German, Chinese) and place of residence; Excluded women had a hysterectomy, bilateral ovariectomy or antioestrogen therapy | - The question whether the differences found are solely cultural or migration-related must be examined in further studies.  - The special experiences and situation in life of migrant women should be taken into particular account by attending physicians during the care and treatment of women in this phase of life. |
| 29 | (Chang & Im, 2015) | Hispanic and Asian women in the United States of America | To develop a theoretical model to explain the relationships between immigration transition and midlife women’s physical activity and test the relationships among the major variables of the model | Quantitative; Questionnaire to answer six questions on sociodemographic characteristics, including  age, education, marital status, family income (difficulty in paying for basics such as food, housing, and clothing), number of children, and employment status | 250;  From a secondary analysis with data from 127 Hispanic  women and 123 non-Hispanic Asian women in a national Internet study; Included women aged 40-60 years | - Immigration transition can play an essential role in influencing health behaviors of immigrant populations in USA  - The non-Hispanic theoretical model can be widely used in nursing practice and research that focus on immigrant women and their health behaviors  - Health care providers need to consider the influences of immigration transition to promote immigrant women’s physical activity  - Because Non-Hispanic Asian women born outside the United States may be affected by their culture of origin, they might be more physically inactive than their U.S.-born counterparts |
| 30 | (Im, Chang, Chee, Chee, & Mao, 2015) | Hispanic, Non-Hispanic Asian, Non-Hispanic African American, Non-Hispanic White in the United States of America | To explore the relationships between immigration transition and depressive symptoms among 1,054 midlife women in the United States | Quantitative;  Questions on background characteristics and immigration transition and the Depression Index for Midlife Women were used to collect the data. | 1054 (316 Non-Hispanic Whites, 255  Hispanics, 250 Non-Hispanic African Americans, and 233 Non-Hispanic Asians); Secondary analysis from two National Internet survey studies that were collected from 2005 to 2013; Included women were midlife women aged 40 to 60 years, literate in English, and reported their ethnic identity as Hispanic, Non-Hispanic Asian, Non-Hispanic African American, or Non-Hispanic White | - Immigrants are more likely to be healthy and resilient and willing and able to respond to possible health hazards due to migration  - Immigrants reported lower numbers of symptoms and less severe symptoms than nonimmigrants  - Health care providers need to consider that self-reported racial/ethnic identity would be a better predictor of ethnic minority midlife women’s depressive symptoms than immigration status in development of their mental health interventions for the population. |
| 31 | (Lee, Wilbur, Chae, Lee, & Lee, 2015) | Chinese, South Asian, Indian, Mexican women in Korea | To investigate the barriers to performing stretching exercise experienced by Korean-Chinese female migrant workers during a community- based 12-week stretching exercise intervention trial. | Qualitative;  Telephone counselling interviews  This study is a secondary data analysis of an existing qualitative data. The qualitative data used in the secondary analysis were semistructured telephone counseling interviews transcripts with 27 Korean-Chinese female migrant workers, which were derived from an original research of a community-based 24 week stretching exercise intervention. | 27;  No inclusion or exclusion criteria | - Participants experienced an average of 2.5 barriers during the study period.  - Intrapersonal barriers included lack of time and lack of motivation, and interpersonal barriers included no family to provide support and also a feeling resistance from coworkers.  - Work-related environmental barriers included frequent job changes, long working hours, lack of rest time, and unpredictable job demands. |
| 32 | (Blumstein, Benyamini, Boyko, & Lerner-Geva, 2016) | Russian women, and Arab Isrealis in Israel | To assess levels of knowledge about  risk factors for heart disease among midlife Israeli women, and to  evaluate the relationship of knowledge to personal risk factors and  vulnerability to heart disease | Quantitative, Face-to-face interviews using a structured questionnaire | 814;  randomly selected from the National Population Registry; Interviews conducted at participant’s homes;  Included women aged 45-64 years, Sampled in three main sub-population groups: Jewish residents born in Israel or who immigrated to Israel up to 1989 (540 participants), Jewish immigrants from Russia who arrived after 1989 (151 immigrants), and Arab Israelis (123 Arabs) | - Wide disparities in knowledge by educational level and between immigrants and long-term residents, after taking into account personal risk factors and education  - Personal risk factors were not significantly related to the knowledge items, except for personal history of cardiovascular disease, which was associated with knowledge about “warning signs of a heart attack” and “family history.” |
| 33 | (Elran-Barak et al., 2016) | Long-Term Jewish Residents, Immigrants from Russia, and Arabs in Israel | To examine cultural differences in Weight status misperception and identify associations between weight perception and weight control efforts among overweight/ obese midlife women in Israel. | Quantitative; structured questionnaire | 814 total: 540 Long-Term Jewish Residents, 151 Immigrants, and 123 Arab women;  Secondary analysis on data from the Women’s-Health-in-Midlife-National-Study that was designed to sample three main sub-population groups in Israel that  represented over 95 % of women aged 45–64 years living in Israel in the early 2000s; Sampled women were contacted by telephone and interviews were conducted at participants’ homes; Included women were limited to overweight/ obese participants  from the WHiMNS (BMI over or equal to 25) | - Health care providers are encouraged to pay attention to overweight/obese women who misperceive their weight status  - Overweight/obese women are more likely to consume unhealthy foods and to be at higher risks of suffering from medical complications associated with obesity  - Women who misperceived their weight status had less  education, lower income, and higher unemployment rates |
| 34 | (Strezova et al., 2017) | Macedonian women in Australia | To explore the attitudes to, and experience of, menopause among Macedonian women living in Australia, including attitudes and responses to hormone therapy (HT) and complementary therapies, as well as  related psycho-sexual, relationship and other midlife issues | Qualitative;  study was based on seven unstructured, nondirective group discussions. | 81; The nondirective, ‘‘affinity’’ group discussion method  involves a naturally existing social group (typically, 5-8 friends, neighbors, work colleagues) meeting together in a familiar setting such as a home, club or workplace, or  wherever the group felt most comfortable;  Included women were 45-75 years | - Culturally determined attitudes appear to affect the perception and experience of menopause.  - Other influencing factors include migration, women’s roles, marital status, religion, use of herbal and traditional  remedies, social situation, access to information, knowledge and experience of menopausal symptoms. |
| 35 | (Salma, Hunter, Ogilvie, & Keating, 2018) | Arab Muslim women in Canada | To examine the results of an interpretive descriptive study about Arab immigrant women’s experiences of practicing stroke prevention | Qualitative;  Semi-structured interviews | 16; Recruited from Religious centres; All participants chose to be interviewed at home except one who  was interviewed at a mosque; Included women were 45-75 years, living in the community, combination of stroke risk factors | - Economic status, access to transportation, language fluency, life stressors, and personal coping strategies influenced Arab women’s ability to manage personal health  - Factors that enhanced personal agency were having strong faith, a good command of the English language, and being able to drive (including access to a car).  - The factors that influenced personal health management in women’s narratives were the result of their social positions as women, immigrants, and visible minorities in Canada.  - During periods of stressful transition, such as being diagnosed with a chronic health condition or experiencing a negative health event, many women in the study became increasingly vulnerable.  - Women who were more educated, had higher economic prosperity, and were more fluent in English were less vulnerable and were able to navigate the healthcare system and utilize health-supporting resources. |
| 36 | (Im, Ko, Lee, Chee, & Chee, 2019) | Hispanic, Non-Hispanic Asian, Non-Hispanic African American, Non-Hispanic White in the United States of America | To explore the associations of immigration to sleep-related symptoms among four major racial/ethnic groups of 1,054 midlife women in the United States. | Quantitative;  The instruments included questions on background characteristics, health and menopausal status, immigration transition, and the Sleep Index for Midlife Women. | 1054 (316 Non-Hispanic Whites, 255  Hispanics, 250 Non-Hispanic African Americans, and 233 Non-Hispanic Asians); Secondary analysis from two National Internet survey studies that were collected from 2005 to 2013; Included women were midlife women aged 40 to 60 years, literate in English, and reported their ethnic identity as Hispanic, Non-Hispanic Asian, Non-Hispanic African American, or Non-Hispanic White  This study uses the same participant population as (Im, Chang, Chee, Chee, & Mao, 2015) | - Immigrants reported smaller number and lower severity scores of cognitive symptoms compared with nonimmigrants.  - Self-reported racial/ethnic identity would explain cognitive symptoms better than other variables related to immigration transition.  - Health-care providers need to consider self-reported racial/ethnic identity as a factor significantly related to sleep-related symptoms during the menopausal transition. |
| 37 | (Ussher, Hawkey, & Perz, 2019) | Afghan, Indian (Punjab), Iraqi, Somalian,  South Sudanese, Sri-Lankan (Tamil), Sudanese and various south American  (Latina) women in Australia and Canada | To examine the construction and  experience of menopause among migrant and refugee women  who had settled in Australia or Canada in the last 10 years | Qualitative;  84 individual interviews and 16 focus groups comprising  85 participants were conducted | 169; Included women were migrant and refugee women 18 years and over who had settled in Sydney, Australia or Vancouver, Canada in the last 10 years, from Afghanistan, India (Punjab), Iraq, Somalia, South Sudan, Sri Lanka (Tamil), Sudan and various South American (Latina) backgrounds | - Thematic decomposition identified three discursive themes: Menopause as the Age of Despair; a Discourse of Silence and Secrecy; and Menopause as a Life Stage – or when Life Starts.  - Negative constructions of menopause, associated with silence and secrecy, were evident across different cultural groups, with implications for women’s positioning and experience of menopausal change and embodiment.  - Resistance to negative discourse was also evident. This was primarily associated with having received menopause education and more open communication about menopausal change, suggesting that education and health information can facilitate affirming aspects of menopause. |
